# Overestimated climate warming and climate variability due to spatially homogeneous CO_2_ in climate modeling over the Northern Hemisphere since the mid-19^th^ century

**DOI:** 10.1038/s41598-019-53513-7

**Published:** 2019-11-22

**Authors:** Xuezhen Zhang, Xiaxiang Li, Deliang Chen, Huijuan Cui, Quansheng Ge

**Affiliations:** 1 Key Laboratory of Land Surface Pattern and Simulation, Institute of Geographical Sciences and Natural Resources Research, Chinese Academy of Sciences, Beijing, 100101 China; 2 Regional Climate Group, Department of Earth Sciences, University of Gothenburg, Gothenburg, 40530 Sweden; 3 University of Chinese Academy of Sciences, Beijing, 100049 China

**Keywords:** Projection and prediction, Climate and Earth system modelling

## Abstract

Since the mid-19^th^ century, the global atmospheric CO_2_ concentration (ACC) has increased dramatically due to the burning of fossil fuels. Because of unequal population growth and economic development among regions, the ACC increases possess strong spatial variability. Particularly, the increase in ACC has been larger in the mid-latitudes of the Northern Hemisphere (NH) than that at high- and low-latitudes. It is widely accepted that the ACC increase is the main reason for climate change, but the potential impacts of its spatial distribution on the climate system remain unclear. Therefore, we carried out two groups of 150-year experiments with the Community Earth System Model (CESM), using both spatially inhomogeneous (hereafter the SIC experiment) and homogenous (hereafter the SHC experiment) ACC increases in their settings. We found that the models’ divergences occurred over the NH mid-latitudes, the Arctic and the western part of the tropical Pacific. SHC overestimated (underestimated) climate warming over the Artic (mid-latitudes), which may be induced by the intensified westerly and weakened meridional heat exchange between mid- and high latitudes in the NH. Over the tropical Pacific, the overestimation of climate warming may be induced by intensified Walker circulation coupled with the La Niña climate mode. For the entire NH, relative to SIC, SHC overestimated the climate warming from 1850 to 1999 by ~10%. Meanwhile, the SHC experiment also overestimated the interannual variabilities in temperature and precipitation, resulting in more serious extreme events. These findings suggest that human contributions to climate warming and increased extreme events since the industrial revolution may be overestimated when using a spatially homogenous ACC.

## Introduction

Since the industrial revolution, it has been well accepted that the most important anthropogenic impact on global climate change is through greenhouse gas (GHG) emissions, most of which is accounted for by CO_2_ derived from fossil fuel consumption. The anthropogenic emissions of CO_2_ led to a radiative forcing of 1.68+/−0.35 W m^−2^ in 2011 relative to 1750, which is much larger than any other anthropogenic forcing^1^. Moreover, due to the great demand of economic development for fossil fuel consumption, human-induced CO_2_ emissions will further increase in future decades^2^. Therefore, it is crucial to study the impacts of human-induced CO_2_ emissions on the earth’s climate to quantify human influences and improve future climate change predictions. However, in current studies of climate modeling, anthropogenic CO_2_ is usually treated as a well-mixed greenhouse gas, meaning that the atmospheric CO_2_ concentration (ACC) is taken as globally homogeneous^3–5^. However, the reality is that the ACC possesses strong spatial variability, which is characterized by high ACC values over the mid-latitudes of the Northern Hemisphere, and low ACC values over the Southern Hemisphere^6,7^. Therefore, the heterogeneity in CO_2_ emissions and the ACC, should be taken into consideration in climate modeling.

With the release of the Coupled Model Intercomparison Project phase 5 (CMIP5), some earth system models have had the ability to depict the spatial variability in the ACC through implementing human-induced CO_2_ emission flux and biogeochemical modules^8,9^. However, the implications of the spatial variability in the ACC in climate changes are seldom studied. Recently, Stuecker *et al*.^10^ reported that the degree of polar amplification depends strongly on the location of CO_2_ forcing, and the extrapolar forcing makes a negligible contribution to polar amplification, which suggests that different spatial distributions of the ACC may induce different climatic effects. In addition, Navarro *et al*.^11^ reported that a spatially heterogeneous ACC due to human CO_2_ emissions may enhance the realism of global climate modeling by implementing a parameterization of human-induced CO_2_ emissions in CESM. Their study indicates that the spatial variability in the ACC due to the human consumption of fossil fuels may have important climatic effects.

In this study, we attempt to determine the climatic effects of ACC increases with spatial variability due to the human consumption of fossil fuels since the Industrial Revolution, and hence, it is expected to improve our understanding of the roles of humans in climate change. We performed one control experiment (hereafter CTL), which kept the ACC constant, and two groups of sensitive experiments (hereafter SIC and SHC, respectively), which focused on anthropogenic CO_2_ emissions to show the impacts of the anthropogenic ACC increase with spatial variability on climate change.

**Figure 1:**
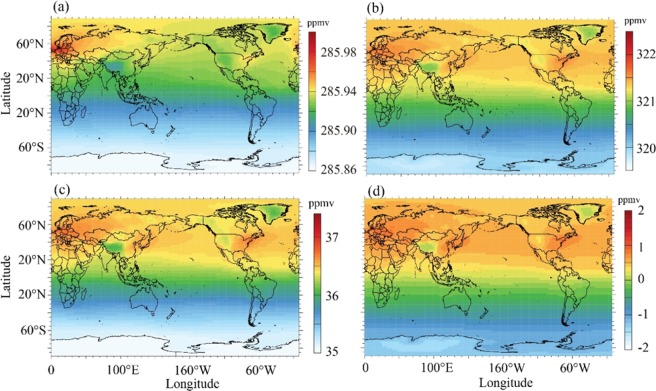
(**a**) Spatial patterns of mean atmospheric CO_2_ concentration of 1850–1899 in SIC. (**b**) Same as (**a**) but for 1950–1999. (**c**) The difference in atmospheric CO_2_ concentration between 1850–1899 and 1950–1999 (1950–1999 minus 1850–1899) in SIC. (**d**) The differences of atmospheric in CO_2_ concentration increments (1950–1999 minus 1850–1899) between SIC and SHC (SIC minus SHC).

In the SIC experiment, the ACC is specified as globally heterogeneous, and associated with human emissions. As shown in Fig. 1a, in 1850–1899, there was a higher ACC in western Eurasia, which was higher by ~0.06 ppmv than other regions in the NH. In the second half of the 20^th^ century, i.e., 1950–1999, the mean ACC over the NH was much higher than that in 1850–1899; meanwhile, the spatial variability in the ACC was intensified, reaching as high as ~4 ppmv between the highest and lowest ACC values (Fig. 1b). There was a high ACC belt across mid-latitudes of the NH with three hot spots of high ACC values, which were in western Europe, East Asia and North America, where high emissions occurred (Fig. S1d). The ACC increments from 1850–1899 to 1950–1999 maintained approximately the same spatial pattern as the spatial pattern of the ACC in 1950–1999 (Fig. 1c). The increments in the ACC were characterized by high values across the mid-latitude belt of the NH with three hot spots, i.e., western Europe, East Asia and North America. This is because the spatial variability in the ACC in 1950–1999 was much larger than that in 1850–1899. The spatial variability in the ACC increments was thereby dominated by that in 1950–1999.

Unlike the SIC experiment, the ACC in the SHC experiment was prescribed as the globally homogeneous, and was set to the global mean of the SIC experiment. This configuration ensures that there are exactly the same global mean ACC for each year, and hence, the simulation differences between SIC and SHC would be induced by the spatial variability in the ACC in SIC. Due to this configuration in SHC, the increments of the ACC from 1850 to 1999 were globally homogeneous. As a result, in comparison to the increments of the ACC in SHC from 1850 to 1999, the increments in SIC were higher over some regions and lower less over other regions. As shown in Fig. 1d, the increments over the three hot spots, which are western Europe, East Asia and North America, were higher by ~1 ppmv in SIC than those in SHC.

**Figure 2:**
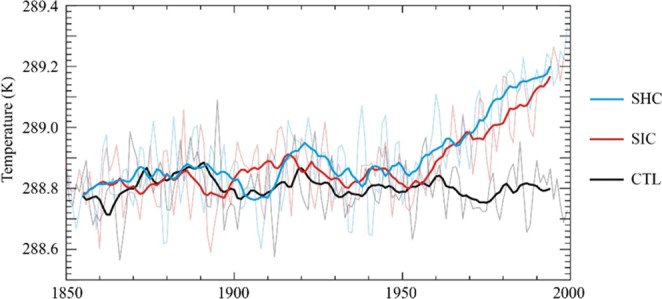
Ensemble mean annual temperature changes over the Northern Hemisphere from 1850 to 1999 for the CTL, SIC, and SHC experiments, respectively (bold line denotes the 11-year moving average).

Figure 2 shows that the annual temperature of the Northern Hemisphere (NH) exhibited a horizontal linear trend from 1850 to 1999 (CTL in Fig. 2) in the absence of anthropogenic emissions and upward trends with a slope of 0.19 K per century (*p* < 0.001) and 0.22 K per century (*p* < 0.001), respectively, for SIC and SHC, due to ACC increases from 284.7 ppmv to 360 ppmv induced by anthropogenic emissions. Moreover, the warming climate mainly occurred in the second half of the 20^th^ century, which is highly synchronized with ACC increases. The NH annual temperature responds to the ACC approximately linearly with a response sensitivity of approximately 0.61 K per 100 ppmv and 0.69 K per 100 ppmv, in the SIC and SHC experiments, respectively. This is consistent with existing modeling-based studies, most of which have demonstrated that the transient sensitivity of the NH annual temperature to ACC ranges from 0.35 K per 100 ppmv to 0.88 K per 100 ppmv^1^.

Although the global mean atmospheric CO_2_ concentration remains the same, the long-term slope in the NH temperature of SHC is slightly larger than that of SIC. Moreover, the mean temperature evolutions in the SIC and SHC experiments can be fairly different over shorter periods. We see this as strong evidence that the spatial variability in the ACC can affect estimated warming. As Fig. 2 shows, the warming climate strength of the SHC experiment was stronger than that of the SIC experiment. In the SHC experiment, the mean annual temperature of 1950–1999 was higher by 0.20 K (±0.04, 95% confidence interval) than that of 1850–1899. Meanwhile, in the SIC experiment, the warming climate strength was 0.18 K (±0.04, 95% confidence interval), which is slightly lower than that in SHC. Moreover, the higher warming climate in the SHC experiment existed through the four seasons (Fig. S4), which may suggest that the SHC simulation overestimates NH climate warming.

**Figure 3:**
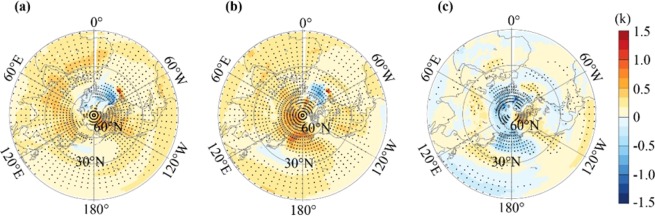
(**a**,**b**) Spatial variability in climate warming (1950–1999 minus 1850–1899) simulated by the SIC (**a**) and SHC (**b**) experiments. (**c**) The differences in climate warming between SIC and SHC (SIC minus SHC). Black symbols are significant at the confidence level of 0.1.

By examining the spatial details, we found that the SIC and SHC experiments produced different spatial patterns of climate warming over the NH (Fig. 3), even opposite patterns in some places. In the SIC experiment, a warming belt occurred over Africa-Eurasia and North America. In Africa and western Asia, the warming belt mainly occurred at 30°N, shifting northward to the mid-latitudes, mostly to 40°N-60°N in central-eastern Asia and to high latitudes at 60°N in North America. However, continuing northward to the northern Pacific and Atlantic, climate warming was much weaker than that of the nearby land. Over the tropical Pacific, climate warming was weaker in the western area than in the eastern area. However, in the SHC experiment, warming occurred extensively over land, and the strongest warming occurred over the northern Pacific and Arctic. Moreover, in the tropical Pacific, the warming was stronger in the western area than in the eastern area, which is the reverse of that of the SIC experiment.

**Figure 4:**
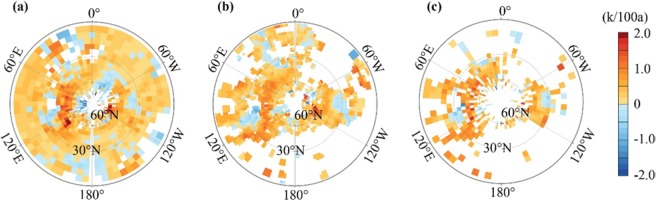
Ground-measured climate warming rate in the 20th century represented by the CRU (**a**, Climatic Research Unit), GISS (**b**, Goddard Institute for Space Studies), and GHCN (**c**, Global Historical Climatology Network) dataset.

Due to the inclusion of only anthropogenic CO_2_ forcing rather than full forcing, neither SIC nor SHC could reproduce the exact spatial variability of climate warming for the 20^th^ century, as illustrated by ground measurements (Fig. 4). Nevertheless, the SIC experiment seemed to match with the ground measurements better than the SHC. Ground measurements highlight that there was strong climate warming occurring in the mid-latitudes of Eurasia, likely shifting northward to the high latitudes. Furthermore, there was relatively weaker climate warming over the northern Pacific. Such spatial variability is well reproduced by SIC, while it is not reproduced by SHC. This shows that modeling with observed spatially variable increases of the ACC more realistically reproduces reproduce the main characters of observed spatial variability of climate warming, while modeling with spatially homogeneous ACC is unable to do this. This finding is consistent with that of Navarro *et al*.^11^ Therefore, we have reasons to believe that SHC overestimates climate warming mainly over the Arctic area and the low latitudes, with the exception of the tropical eastern Pacific, and underestimates warming across the mid-latitudes of land (Fig. 3).

**Figure 5:**
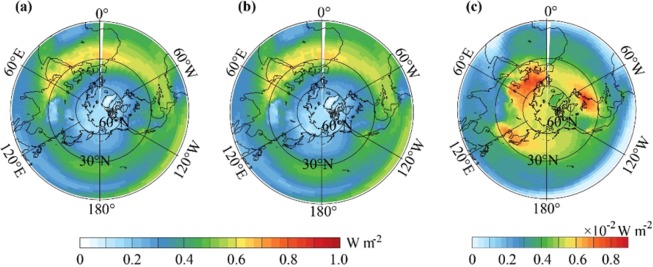
(**a**,**b**) Spatial variability in radiative forcing caused by CO_2_ increase (1850–1899 minus 1950–1999) simulated by the SIC (**a**) and SHC (**b**) experiments. (**c**) Differences in the spatial variability of radiative forcing between SIC and SHC (SIC minus SHC).

To determine the reasons for the different climate warming from 1850 to 1999 between the SIC experiment and SHC experiments, we examined the radiative forcing (RF) induced by ACC increments in SIC and SHC. Figure 5a,b show that the both SIC and SHC shared approximately the same spatial pattern of RF and are dominantly characterized by markedly high RF over the mid- to low latitudes and low RF over the equatorial and polar areas. Such spatial patterns of RF led by CO_2_ increments generally consistent with the findings of existing studies, such as Huang *et al*.^12,13^. This result suggests that the global spatial pattern of RF led by CO_2_ is not determined mainly by the local ACC. However, as shown in Fig. 5c, differences still exist between SIC and SHC. In comparison to SHC, SIC exhibits high RF over the mid- to high latitudes, with three particular hot spots, i.e., Europe, East Asia and eastern North America, which correspond to the three hot spots of CO_2_ increments. This indicates that the RF differences between SIC and SHC are derived from local ACC differences. This result demonstrates that the climatologic spatial pattern of RF led by CO_2_ increment is not determined mainly by the local ACC, while in the context of the climatologic background, RF could be modified by local ACC variations.

The RF is a good proxy indicator for climate warming. The extensive positive RF over the NH shown in Fig. 5a,b represent land-atmosphere systems that hold more energy and, hence, explain the differences in climate warming, as shown in Fig. 2. However, it is distinguished that the temperature has not linearly responded to the local RF. First, in SIC or SHC, the spatial patterns of climate warming are mismatched with the spatial patterns of RF. Second, there is likely more RF over the NH in SIC than in SHC (Fig. 5c), but the NH climate warming is weaker in SIC than in SHC (Fig. 2).

By comparing the spatial pattern of climate warming differences (Fig. 3c) to the spatial pattern of RF (Fig. 5c), we found that the intensified climate warming over western Eurasia and East Asia in SIC may be explained by locally high RF in SIC. Excluding the two regions, climate warming differences become difficult to explain with local RF differences. For instance, the weakened climate warming over the Arctic and tropical western Pacific and the intensified climate warming over the tropical eastern Pacific likely could not be explained by RF differences.

Therefore, the geopotential height and wind vector at a pressure level of 500 hPa were analyzed to understand the underlying mechanism leading to climate warming differences between SIC and SHC. It is noted that there is strong seasonal variability in the NH atmospheric circulation, and among the four seasons, the winter climate warming differences (Fig. S5) are most similar to the annual climate warming differences; this study focuses on winter atmospheric circulation changes.

**Figure 6:**
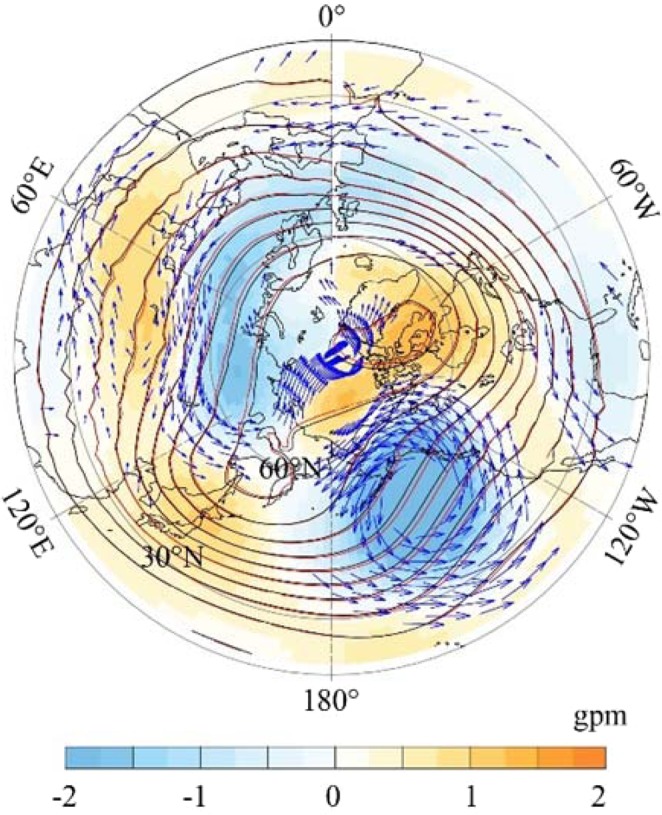
Differences of SIC minus SHC for the changes in geopotential height (color patch) and wind (arrows, only shows the confidence at level of 0.1) at a pressure level of 500 hPa in winter from 1850 to 1999 (mean of 1950–1999 minus mean of 1850–1999). The solid red and black contour lines denote the geopotential height at a pressure level of 500 hPa for 1850–1899 simulated by SIC and SHC, respectively.

Figure 6 shows that SIC exhibits stronger westerlies over the mid- to high latitudes of Eurasia than the SHC. The intensified westerlies could be explained by the higher RF (Fig. 5c) and intensified climate warming over western Eurasia (Figs. 3c and S5). The intensified climate warming, as the result of more RF, may intensify the temperature gradient between mid- and high latitudes distinctly over Eurasia. Therefore, the westerly jet over Eurasia would be intensified^14^ (Fig. 6). The intensified westerly jet suggests that the meridional exchange of heat between mid- and high- latitudes would be weakened. As a result, the climate warming over the mid-latitudes of Eurasia would be strengthened further, whereas that over the high latitudes to Arctic area would be weakened. This result may explain the weakened climate warming over the eastern Arctic in SIC (Fig. 3c).

Meanwhile, due to the intensified zonal wind and the weakened meridional wind over mid- to high-latitude Eurasia, the Rossby wave tends to be straight. As shown in Fig. 6, both the block high over the Ural Mountains and the East Asia trough are weakened. The weakened block high and East Asia trough lead to a weakened East Asian winter monsoon, which would further intensify climate warming in East Asia. Therefore, both local RF induced by more ACC and a weakened East Asian winter monsoon may explain the stronger climate warming in SIC, as shown in Fig. 3c.

Following the Rossby wave dispersion theory proposed by Ye H^15^, the weakened Ural high and East Asian trough, as the upstream components, would lead to a weakened North American high^16^, which is a downstream component. Due to the weakened North American high, the northward movements of warm air in the mid-latitudes of the Pacific Ocean would be blocked; as a result, the climate warming in Alaska and the northern Pacific in the SIC experiment is weakened. Meanwhile, as the lower reach of the westerlies, the North American trough would also be weakened. Both the weakened North American high and North American trough would not favor the southward movement of cold air in eastern North America. As a result, the climate warming in the SIC experiment would be stronger in central and eastern North America than in the SHC experiment.

**Figure 7:**
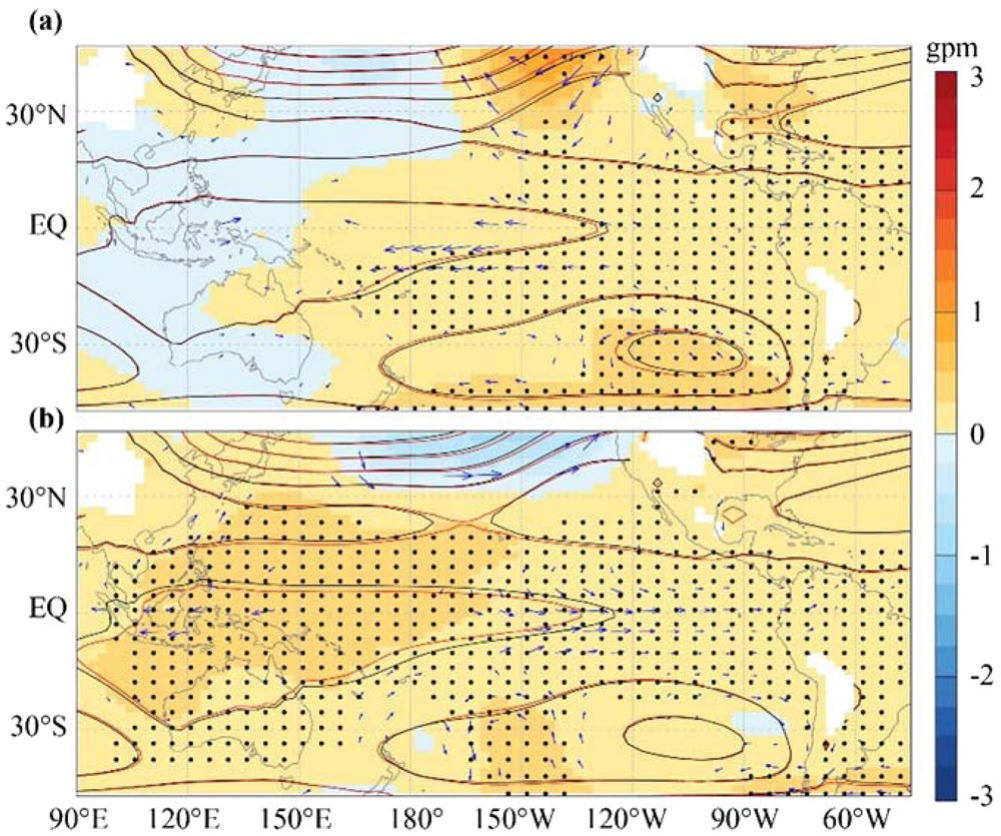
(**a**,**b**) Geopotential height at 850 hPa pressure level for 1850–1899 (solid blue lines), 1950–1999 (solid red lines), their differences (color patch; 1950–1999 minus 1850–1899; Black dots denote significance at the confidence level of 0.1), and wind vector differences (arrows, only significant areas at the confidence level of 0.1 are shown) for the SHC (**a**) and SIC (**b**) experiments.

Figure 6 also shows the approximately positive phase of the Pacific/North American teleconnection (PNA) pattern, which is characterized by the above normal geopotential heights over the western U.S. and below normal geopotential heights over the southeastern U.S. and the North Pacific^17^. The positive phase of PNA is usually accompanied by the El Niño mode in the tropical eastern Pacific, which is characterized by a cold anomaly in the tropical western Pacific and a warm anomaly in the tropical eastern Pacific^18^. The tropical Pacific climate warming differences between SIC and SHC are indeed very similar to the El Niño mode (Fig. 7).

Figure 7 shows the simulated changes in geopotential height and wind vectors at a pressure level of 850 hPa induced by atmospheric CO_2_ concentration increases from 1850 to 1999. Here, the results of the SIC and SHC experiments present opposite patterns of geopotential height and wind anomalies over the tropical Pacific. In the SIC experiment, the ACC increase caused a larger lifting of geopotential height over the western tropic Pacific and a smaller lifting over the eastern tropic Pacific. As a result, there was a westerly wind anomaly that partly offsets the climatologic tropic easterly wind. Such an anomaly of circulation matches well with the El Niño-like mode, in which there is a weakened tropical easterly and warm (cold) anomaly eastern (western) tropic Pacific. Because the tropical easterly moves sea surface warm water from east to west climatologically, the western tropical Pacific is warmer than the eastern tropical Pacific. Once the tropical easterly weakens, the movement of sea surface warm water from east to west weakens. Hence, there would be a warm (cold) anomaly in the eastern (western) tropical Pacific. This pattern is almost reversed in the SHC experiment, which is characterized by a larger (smaller) lifting of geopotential height over the eastern (western) tropical Pacific and an easterly wind anomaly. The easterly wind anomaly intensifies the climatologic tropic easterly wind. Such a circulation anomaly matches well with the La Niña-like mode, which may result in a warming (cooling) anomaly in the western (eastern) tropical Pacific. These findings may provide an explanation for the intensified climate warming in the eastern tropical Pacific and weakened climate warming in the western tropical Pacific in the SIC experiment. Such an El Niño-like mode of climate warming difference may be associated with the abovementioned positive phase of the PNA pattern.

The above analysis leads to the conclusion that although the exact same global mean atmospheric CO_2_ concentration was used for both the SIC and SHC experiments, the spatial heterogeneity in atmospheric CO_2_ concentration does affect climate warming strength and atmospheric circulation. Forcing by spatially heterogeneous atmospheric CO_2_ concentrations yielded weakened Northern Hemisphere climate warming, particularly over the Arctic and western tropic Pacific. Meanwhile, there was intensified climate warming in the mid-latitudes and eastern tropic Pacific. The possible factors leading to climate warming strength differences vary geographically. The positive RF induced by a higher ACC over mid- and high-latitude Eurasia may be crucial forcing. This positive RF over the mid- to high latitudes of Eurasia may not only lead to local warming but may also intensify the westerly jet and weaken the block high over the Ural Mountains. Then, the large-scale wave processes over the mid- to high-latitude NH would be weakened, and meridional heat exchange would also be weakened; as a result, the climate warming over the polar area and mid- to high latitudes would be modified. Over the tropical Pacific, atmospheric circulation due to ocean-atmosphere interactions dominated the climate warming differences between the SIC and SHC experiments.

**Figure 8:**
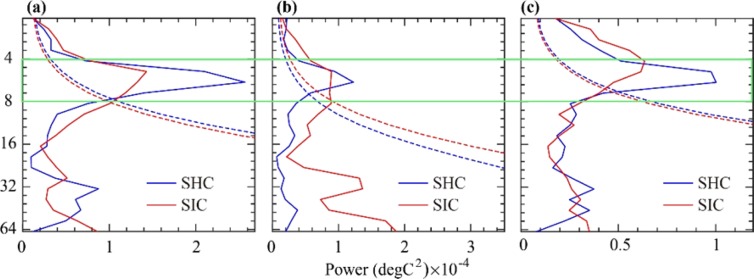
(**a**–**c**) Spectrum profiles of annual precipitation simulated by the SHC and SIC experiments for high latitudes (**a**), mid latitudes (**b**), and low latitudes (**c**). The dashed line denotes the 95% confidence level.

Unsurprisingly, the simulated precipitation variations were also different between the two sensitivity experiments. Figure 8 shows the peak of spectral powers occurring in a period of 4–8 years. Moreover, the locations of the peaks are approximately the same in both SHC and SIC experiments. This suggests that annual precipitation variations produced by both experiments were consistently characterized by large interannual variability. However, the strength of the spectral power of the interannual variability produced by the SHC experiment was much stronger than that of the SIC experiment, particularly for the low- and high latitudes. For the low latitudes, the variability at a scale of 3–7 years accounts for 43.3% of total variance in the SHC experiment, while it accounts for 31.1% in the SIC experiment. For the high-latitudes, the variability at a scale of 3–7 years accounts for 65.0% of the total variance in the SHC experiment, while it accounts for 46.9% in the SIC experiment. This finding suggests that the SHC experiment may overestimate the interannual variability of precipitation over the NH, implying that the strength and frequency of extreme hydroclimate events may be overestimated by the SHC experiment.

Overall, we have demonstrated that the use of spatially homogenous atmospheric CO_2_ concentrations in climate modeling may essentially exert impacts on climate change simulations. Therefore, existing studies with spatially homogenous atmospheric CO_2_ concentrations may have overestimated the Northern Hemisphere climate warming strength and climate variability since 1850, particularly since 1950. They also underestimated the temperature gradient from mid-latitudes to high-latitudes particularly in Eurasia, by overestimating (underestimating) climate warming over high latitudes (mid latitudes). As a result, other circulations, such as the westerly jet and Arctic Oscillation, which are closely related to mid- to high-latitude temperature gradients, might be more or less mistakenly simulated.

Meanwhile, there are still several relevant issues that warrant discussion. Land and ocean biospheres play very important roles in atmospheric CO_2_ concentrations, such as seasonal dynamics^19–21^. However, these factors were absent in our abovementioned experiments. With fewer factors influencing atmospheric CO_2_ concentration, the absence of these processes may lead to a spatially smoothed atmospheric CO_2_ concentration, which is explained in our experiment (Fig. S3). Due to the weakened spatial variability of CO_2_ concentration, the differences in climate warming strength between the SIC and SHC experiments may be weakened. Therefore, differences in the actual climate warming strength may be even larger than our illustrations suggest.

Additionally, atmospheric CO_2_ concentration may also exert an impact on terrestrial and ocean biospheres and, hence, on surface albedo; as a result, climate change would be modified^22–25^. However, due to the absence of a biogeochemical cycle in our modeling, such modifications of atmospheric CO_2_ concentration dynamics on climate change were absent. Thus, our study has not comprehensively considered all the impacts of atmospheric CO_2_ concentration dynamics on climate changes. Moreover, historical climate change was combined with the effects of external forcing and internal variability. In comparison to the real complex process of the earth climate system, we only focused on the radiation-related impacts of fossil fuel CO_2_ emissions, and the modeled climate is not comparable to observations. To deepen our understanding of the impacts of spatially heterogeneous atmospheric CO_2_ concentrations, much more explicit modeling studies are necessary.

## Method

We used the CESM version 1.0.3 in this study for climate warming simulation. As a successor to the Community Climate System Model, version 4 (CCSM 4), the CESM incorporates new earth system simulation capabilities^26^, becoming a flexible and extensible community tool to investigate diverse sets of earth system interactions across multiple temporal and spatial scales. Here, we used the CESM with active atmosphere, ocean, land, and sea-ice components. In detail, the Community Atmosphere Model, version 4 (CAM4), the Community Land Model, version 4 (CLM4), Parallel Ocean Program, version 2 (POP2), and the Los Alamos National Laboratory (LANL) Community Ice Code, version 4 (CICE4) were utilized. The atmosphere and land components have grid cells with dimensions of 1.9° (latitude) by 2.5° (longitude), and the ocean and sea-ice components have grid cells with dimensions of approximately 1° (latitude) by 1° (longitude).

In total, we carried out three groups of simulations (Table 1) using the abovementioned model configurations. First, we carried out a control run (CTL) of 1250-years with the initial conditions for 1850 and constant preindustrial external forcing, including a total solar irradiation of 1360.89 Wm^−2^, a CO_2_ concentration of 284.7 ppmv, a CH_4_ concentration of 791.6 ppbv, and a N_2_O concentration of 275.68 ppbv. Meanwhile, in the control run, the atmospheric CO_2_ concentration was prescribed to be globally homogeneous. The first 250 years of the simulation were discarded as spin-up, and the stability of the later 1000-year simulation was verified by intermodel comparisons^27^.

**Table 1:** List of experimental design aspects.

Experiment name	Short name	Time control	CO_2_ concentration
Control run	CTL	1–1250 (model year)	Fixed at 284.7ppmv everywhere
Spatially inhomogeneous CO_2_ runs	SIC runs, members: SIC228, SIC483, SIC630	Branch runs starting from 228^th^, 483^td^, and 630^th^ years, lasting for 150 years	Dynamic CO_2_ concentration with spatial differences, derived from the NCAR dataset^28^
Spatially homogeneous CO_2_ runs	SHC runs, members: SHC228, SHC483, SHC630	Branch runs starting from 228^th^, 483^td^, and 630^th^ years lasting for 150 years	Dynamic but spatially homogeneous CO_2_ concentrations, keeping yearly global mean atmospheric CO_2_ concentration exactly the same as the corresponding SIC runs

Then, as the branch runs of the CTL run, we carried out two groups of sensitivity experiments, namely, spatial inhomogeneous CO_2_ runs (hereafter SIC runs) and spatially homogeneous CO_2_ runs (hereafter SHC runs). Each sensitivity experiment consisted of three members, which are branch runs of the CTL starting at the 228th year, 483th year, and 630th year, and lasting for 150 years. The SIC and SHC runs used the same settings as the CTL run, with the exception of atmospheric CO_2_ concentration.

The dynamic human-induced CO_2_ emission flux used in the SIC and SHC runs was derived from a global historical fossil fuel CO_2_ emission flux estimation dataset for CMIP5 simulations covering the period from 1850 to 2000 provided by NCAR^28^ (available at https://svn-ccsm-inputdata.cgd.ucar.edu/). Here, national CO_2_ emissions were estimated from fossil-fuel burning, gas flaring in oil fields, cement manufacturing, energy production, consumption, and trade (see Andres *et al*.^28^, for details). Thereafter, the gridded annual CO_2_ emissions were created by allocating the national-level CO_2_ emissions to each grid weighted by the grid’s population density. We prescribed a above gridded CO_2_ emissions in the SIC runs. Within CESM, the atmospheric CO_2_ concentration for each model cell and each atmospheric level is calculated through the embedded aerodynamics-based diffusion model in order for the SIC runs to be forced by a heterogeneous atmospheric CO_2_ concentration. In contrast, we prescribed the homogenous yearly dynamic CO_2_ concentration in the SHC runs, where the yearly dynamic global mean CO_2_ concentrations were averaged from that of SIC runs. Therefore, the SHC runs keep exactly the same global mean atmospheric CO_2_ concentration as the SIC runs for each year.

The Parallel Offline Radiative Transfer (PORT) model, as a CESM tool for the diagnosis of radiative forcing (RF), was used to calculate the RF of the increased atmospheric CO_2_ concentration. It is isolated from CAM4 in CESM1 and driven by model-generated datasets, including the CAM4-generated data^29^. The PORT model can calculate the instantaneous radiative forcing and stratospherically adjusted radiative forcing. In this study, the instantaneous radiative forcing reported at the top of the atmospheric model was used.

To compute the instantaneous radiative forcing of CO_2_ from the SIC (SHC) experiments, we ran the PORT model using meteorology data from CTL experiments together with the prescribed CO_2_ concentration from the SIC experiments, SHC experiments and CTL. In detail, the PORT model was driven using meteorology data for three segments, i.e., 228–377, 483–632, and 630–779, respectively, from the CTL experiments to represent the same periods as the SIC and SHC sensitive experiments. Then, the mean radiative flux of the three PORT runs with the human-induced ACC in SIC experiments and SHC experiments was contrasted to that forced by the ACC from CTL. These differences were used to determine the radiative forcing.

## Supplementary information

Supporting Informaiton

## Data Availability

The authors declare that the data will be available without restrictions.

## References

[1] IPCC. Climate Change 2013: The Physical Science Basis. Contribution of Working Group I to the Fifth Assessment Report of the Intergovernmental Panel on Climate Change [Stocker, T. F., Qin, D., Plattner, G.-K., Tignor, M., Allen, S. K., Boschung, J., Nauels, A., Xia, Y., Bex, V. and Midgley, P. M. (eds)]. Cambridge University Press, Cambridge, UK and New York, NY, 1535 (2013a).

[2] Rogelj J (2016). Paris Agreement climate proposals need a boost to keep warming well below 2 °C. Nature.

[3] Neale, R. B. *et al*. Description of the NCAR Community Atmosphere Model (CAM 5.0), https://www.ccsm.ucar.edu/models/ccsm4.0/cam/docs/description/cam4_desc.pdf (2012).

[4] Taylor KE, Stouffer RJ, Meehl GA (2012). An overview of CMIP5 and the experiment design. B. Am. Meteorol. Soc..

[5] Wei T (2012). Developed and developing world responsibilities for historical climate change and CO_2_ mitigation. PNAS..

[6] Chahine MT (2008). Satellite remote sounding of mid-tropospheric CO_2_. Geophys. Res. Lett..

[7] Zhou C, Shi R, Liu C, Gao W (2013). A correlation analysis of monthly mean CO_2_ retrieved from the Atmospheric Infrared Sounder with surface station measurements. Int. J. Remote Sens..

[8] Rickard GJ, Behrens E, Chiswell SM (2016). CMIP5 earth system models with biogeochemistry: An assessment for the southwest Pacific Ocean. J. Geophys. Res-Oceans.

[9] Winkler AJ, Myneni RB, Alexandrov GA, Brovkin V (2019). Earth system models underestimate carbon fixation by plants in the high latitudes. Nat. Commun..

[10] Stuecker MF (2018). Porlar amplification dominated by local forcing and feedbcaks. Nat. Clim. Change..

[11] Navarro A, Moreno R, Tapiador FJ (2018). Improving the representation of anthropogenic CO2emissions inclimate models: A new parameterization for the Community Earth System Model (CESM). Earth Syst. Dyn. Discuss..

[12] Huang Y, Tan X, Xia Y (2016). Inhomogeneous radiative forcing of homogeneous greenhouse gases. J. Geophysical Res.-Atmosphere.

[13] Huang Y, Xia Y, Tan X (2017). On the pattern of CO_2_ radiative forcing and poleward energy transport. J. Geophys. Res.-Atmosphere.

[14] Zhang YC, Takahashi M, Guo L (2008). Analysis of the East Asian Subtropical Westerly Jet simulated by CCSR/NIES/FRCGC coupled climate system model. J. Meteor. Soc. Japan..

[15] Yeh TC (1949). On energy dispersion in the atmosphere. J. Meteor..

[16] Huang, R. H. *et al*. Development from the theory of energy dispersion of rossby waves to studies on the dynamics of quasi-stationary planetary waves. *Chinese Journal of Atmospheric Sciences*. **1**, 10.3878/j.issn.1006-9895.1503.14298 (2016).

[17] Domeisen DIV, Garfinkel CI, Butler AH (2019). The teleconnection of El Niño southern oscillation to the stratosphere. J. Geophys. Res-Atmos..

[18] Seager R (2010). Adjustment of the atmospheric circulation to tropical Pacific SST anomalies: Variability of transient eddy propagation in the Pacific-North America sector. Q. J. Roy. Metor. Soc..

[19] Peng S (2015). Benchmarking the seasonal cycle of CO_2_ fluxes simulated by terrestrial ecosystem models. Global Biogeochem. Cy..

[20] Shaw EC, Mcneil BI (2014). Seasonal variability in carbonate chemistry and air-sea CO_2_ fluxes in the southern Great Barrier Reef. Mar. Chem..

[21] Yan H (2016). Seasonal variations of seawater *p*CO_2_ and sea-air CO_2_ fluxes in a fringing coral reef, northern South China Sea. J. Geophys. Res-Oceans..

[22] Hauck J, Völker C (2015). Rising atmospheric CO_2_ leads to large impact of biology on Southern Ocean CO_2_ uptake via changes of the Revelle factor. Geophys. Res. Lett..

[23] Liu S, Zhuang Q, Chen M, Gu L (2016). Quantifying spatially and temporally explicit CO_2_ fertilization effects on global terrestrial ecosystem carbon dynamics. Ecosphere..

[24] Schimel D, Stephens BB, Fisher JB (2015). Effect of increasing CO_2_ on the terrestrial carbon cycle. P. Natl. Acad. Sci. USA.

[25] Sun Z (2018). Spatial pattern of GPP variations in terrestrial ecosystems and its drivers: Climatic factors, CO_2_ concentration and land-cover change, 1982–2015. Ecole. Inform..

[26] Hurrell JW (2013). The community earth system model: a framework for collaborative research. B. Am. Meteorol. Soc..

[27] Zhang X, Wu M, Liu Y, Hao Z, Zheng J (2018). The relationship between the East Asian Summer Monsoon and El Niño-Southern Oscillation revealed by reconstructions and a control simulation for millennium. Quatern. Int..

[28] Andres RJ, Gregg JS, Losey L, Marland G, Boden TA (2011). Monthly, global emissions of carbon dioxide from fossil fuel consumption. Tellus. B..

[29] Conley AJ, Lamarque J-F, Vitt F, Collins WD, Kiehl J (2013). PORT, a CESM tool for the diagnosis of radiative forcing. Geosci. Model Dev..

