# Supplementary material for: Overestimated climate warming and climate variability due to spatially homogeneous CO2 in climate modeling over the Northern Hemisphere since the mid-19th century
Source: Sci Rep. 2019 Nov 22;9:17426. doi: 10.1038/s41598-019-53513-7 (PMC6874654; doi:10.1038/s41598-019-53513-7)
Supplement: Supplementary file 1 — Supporting Informaiton [file 41598_2019_53513_MOESM1_ESM.docx]

Supporting information

Overestimated climate warming and climate variability due to spatially homogeneous CO_2_ in climate modeling over the Northern Hemisphere since the mid-19^th^ century

Xuezhen Zhang^1, 3^, Xiaxiang Li^1, 3^, Deliang Chen^2^, Huijuan Cui^1^, Quansheng Ge^1*^

1 Key Laboratory of Land Surface Pattern and Simulation, Institute of Geographical Sciences and Natural Resources Research, Chinese Academy of Sciences, Beijing 100101, China

2 Regional Climate Group, Department of Earth Sciences, University of Gothenburg, Gothenburg, Sweden

3 University of Chinese Academy of Sciences, Beijing 100049, China

**Correspondence to*: Prof. Quansheng Ge ([geqs@igsnrr.ac.cn](mailto:geqs@igsnrr.ac.cn))

Through 1850-1999, there were three high fossil-fuel CO_2_ emission centers across the mid latitudes of the Northern Hemisphere which are located in Western Europe, the Northeastern United States, and East Asia, respectively (Figure S1). The CO_2_ emission flux was as high as 2.0-4.0 kg m^-2^ s^-1^ in Western Europe and the Northeastern United States. In East Asia, it was much less, only 1.0-2.0 kg m^-2^ s^-1^. Beyond the three hot-spots in the mid latitudes of the Northern Hemisphere, anthropogenic CO_2_ emissions are too weak to be detected.

From 1850 to 1999, the spatial variability of CO_2_ emission strength has changed. In the first 50-years, i.e., 1850-1899, CO_2_ emission strength was generally weak. In comparison to the global mean, there were relatively strong CO_2_ emissions, which was only 0.5-1.5 kg m^-2^ s^-1^, in Western Europe. In the second 50-year period, i.e., 1900-1949, CO_2_ emissions intensified mainly in Western Europe and the Northeastern United States, in which the emission flux reached about 1.4-3.0 kg m^-2^ s^-1^ and 1.0-2.7 kg m^-2^ s^-1^, respectively. In the third 50-year period, i.e., 1950-1999, the CO_2_ emissions intensified extensively over the mid latitudes of the Northern Hemisphere, in particular in Western Europe, the Northeastern US, and Eastern Asia. The emission flux reached as high as 2.5-4.0 kg m^-2^ s^-1^ and 2.3-3.5 kg m^-2^ s^-1^ in Western Europe and the Northeastern US, respectively. As a rising economic regime, Eastern Asia became a new high CO_2_ emission center, in which the emission flux reached 1.5-3.0 kg m^-2^ s^-1^. These figures suggest that CO_2_ emissions increased mostly in the third 50-year period, i.e., 1950-1999. The dominant reason was the extensively strengthened CO_2_ emissions in Western Europe and the Northeastern US, as well as a newly arising high emission center in Eastern Asia.

Atmospheric CO_2_ concentrations increased largely as the result of human fossil-fuel CO_2_ emissions. The SIC experiment simulated that the global mean atmospheric CO_2_ concentration increased from about 285 ppmv in 1850 to nearly 360 ppmv in 1999 (Figure S2). Such an increment matches well with observations, illustrating that atmospheric CO_2_ concentrations increased from 287.73 in 1850 ppmv to 366.36 ppmv in 1999 (Keeling et al., 2005; Meure et al., 2006). In the context of increasing CO_2_ concentrations, Figure S2 shows that the increment of global mean atmospheric CO_2_ concentration was low before 1900, while thereafter it intensified. In particular, since the mid-20^th^ century, there was a distinguished increment. Such a temporal variability of increment matches well with observations. These findings suggest that the SIC experiment reproduced well the temporal variability of atmospheric CO_2_ concentrations over the last 150 years.

Due to spatially different fossil-fuel CO_2_ emission strengths, the SIC experiment presents that atmospheric CO_2_ concentration exhibits spatial variability. Figure 1b（in main text） shows an increasing tendency from the Southern Hemisphere to the Northern Hemisphere, with a peak occurring in the mid latitudes of the Northern Hemisphere, corresponding to the high CO_2_ emissions. Meanwhile, within the mid latitudes of the Northern Hemisphere, the atmospheric CO_2_ concentration also exhibits spatial differences, characterized by the high concentrations over Europe, the Eastern United States, and East Asia, with lower concentrations over the Tibetan Plateau and North Pacific. The abovementioned zonal and meridian profile patterns derived from SIC experiment are similar to those from observations (Figure S4). Meanwhile, Figure 1d (in main text) shows that the spatial pattern of the increasing trend of atmospheric CO_2_ concentration from 1850 to 1999 matches well with that of mean atmospheric CO_2_ concentration.

It appears that the spatial variability of atmospheric CO_2_ concentrations derived from the SIC experiment is much weaker than that from observations (Figure S4). Observation shows that in the context of the increasing tendency from the Southern to Northern Hemisphere, there is sub-scale of variability in the zonal profile, as well as the meridian profile. For instance, from the equator to ~50°N, the SIC experiment presents the atmospheric CO_2_ concentration difference as less than 0.5 ppmv, while observations pose it as larger than 2 ppmv. On the meridian profile, the range of atmospheric CO_2_ concentration is less than 0.5 ppmv in the SIC experiment, while it is about 0.8 ppmv in observation. This finding suggests that the SIC experiment could reproduce the general pattern of zonal and meridian profiles but is unable to precisely reproduce the spatial variability from observations. This shortage in the SIC experiment may be due to the simplification of its aerodynamic diffusion model. The model simplification works like a lower-pass filter which removes the small scale of variability. Additionally, the mean zonal and meridian profiles refer to different temporal scales, which are 1950 to 1999 for the SIC experiment and 2002 to 2011 for observations. Such different temporal scales may also be in part responsible for the different variability between the SIC experiment and observations.


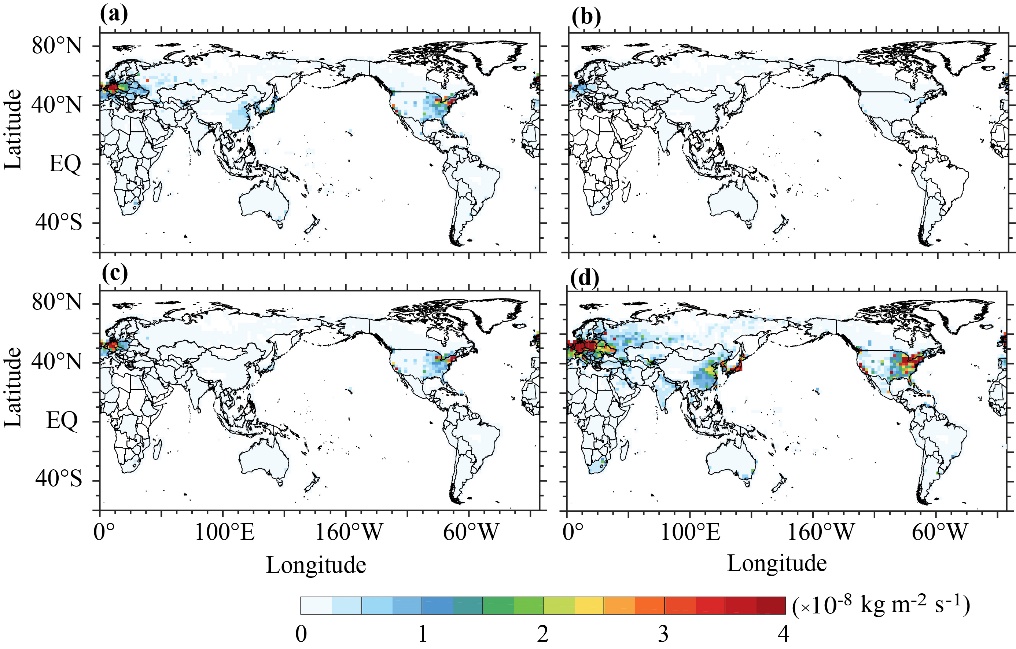


**Figure S1** **a, b, c, d**, Mean fossil-fuel CO_2_ emission flux (Andres et al., 2011) for 1850-1999 (**a**), 1850-1899 (**b**), 1900-1949 (**c**), and 1950-1999 (**d**), respectively


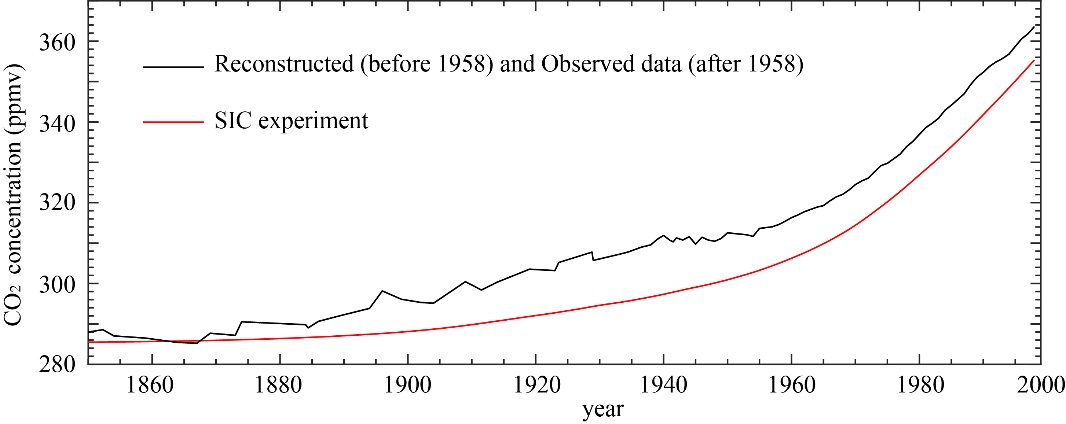


**Figure S2** Global mean atmospheric CO_2_ concentration simulated by the SIC experiment forced by fossil-fuel CO_2_ emissions and the reconstructed/instrument-measured CO_2_ concentration (Keeling et al., 2005)


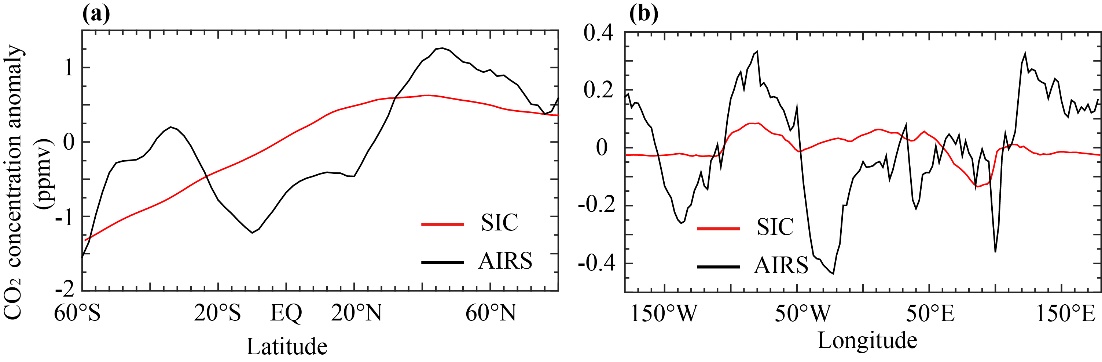


**Figure S3** **a, b,** Zonal mean profiles (**a**) and Meridian mean profiles (**b**) of atmospheric CO_2_ concentration for 1950-1999 from the SIC experiments and 2002-2011 from Atmospheric Infrared Sounder (AIRS) measurements on board Aqua satellite (Zhang et al., 2015)


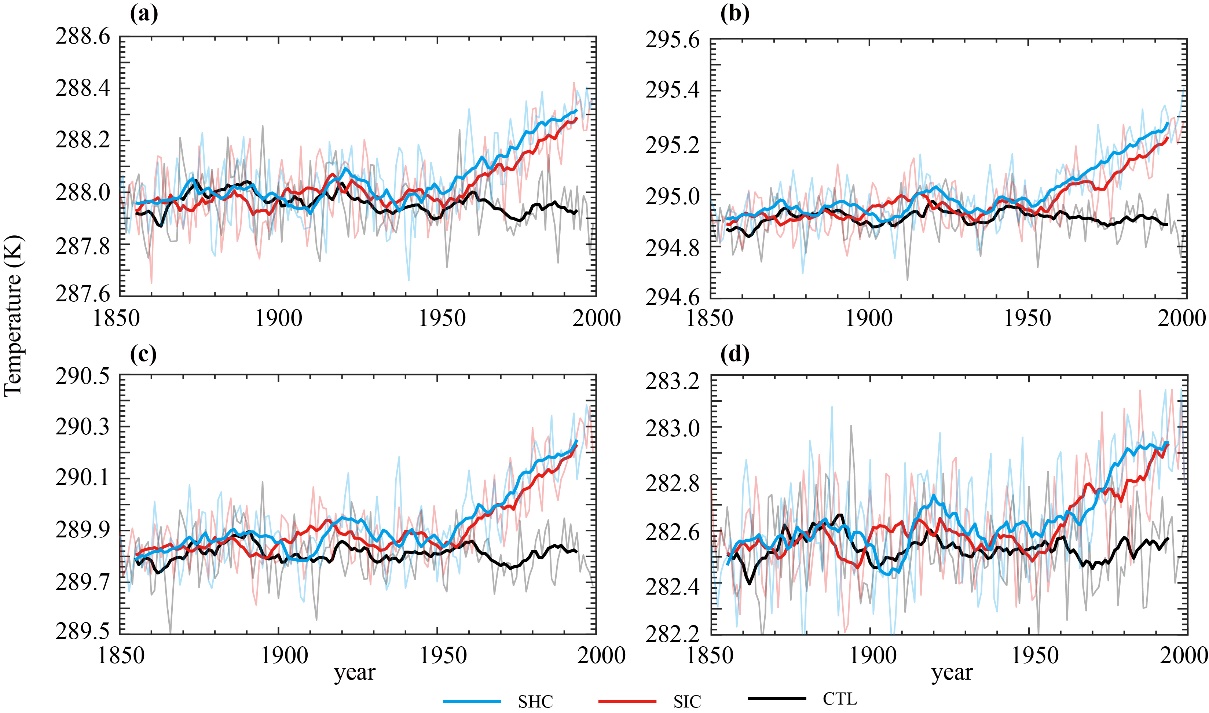


**Figure S4** Ensemble mean seasonal (**a,** spring; **b,** summer; **c,** fall; **d,** winter) temperature changes over the Northern Hemisphere from 1850 to 1999 for the CTL, SIC, and SHC experiments, respectively (bold line denotes 11-year smooth)


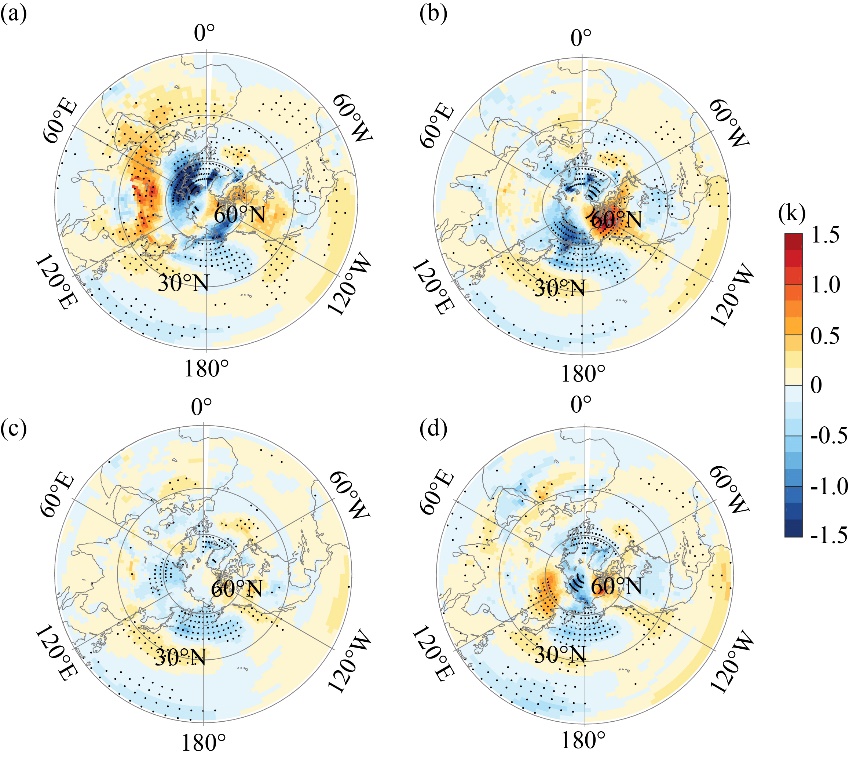


**Figure S5** **a,** Differences of SIC minus SHC for the climate warming in winter from 1850 to 1999 (mean of 1950-1999 minus mean of 1850-1999). b, c, d same with a, but for spring, summer and autumn. Black symbols are significant at the confidence level of 0.1.

**References**

Keeling, C. D., Piper, S. C., Bacastow, R. B., Wahlen, M., Whorf, T. P., Heimann, M., and Meijer, H. A., 2005. Atmospheric CO_2_ and ^13^CO_2_ exchange with the terrestrial biosphere and oceans from 1978 to 2000: Observations and carbon cycle implications, in: A history of atmospheric CO_2_ and its effects on plants, animals, and ecosystems, Springer, New York, NY, USA, 83-113.

Meure, M.F., Etheridge, D., Trudinger, C., Steele, P., Langenfelds, R., Ommen, T.V., Smith, A., Elkins, J., 2006. Law Dome CO_2_, CH_4_ and N_2_O ice core records extended to 2000 years BP. Geophys. Res. Lett. 33(14), 70-84.

Andres, R.J., Gregg, J.S., Losey, L., Marland, G., Boden, T.A., 2011. Monthly, global emissions of carbon dioxide from fossil fuel consumption. Tellus B 63(3), 309-327.
